# Lipoxygenase Pathway Mediates Increases of Airway Resistance and Lung Inflation Induced by Exposure to Nanotitanium Dioxide in Rats

**DOI:** 10.1155/2014/485604

**Published:** 2014-02-17

**Authors:** Jyu-Feng Lee, Shu-Ping Tung, David Wang, Diana Yuwung Yeh, Yao Fong, Yu-Chung Young, Fur-Jiang Leu

**Affiliations:** ^1^Department of Nursing, St. Mary's Medicine Nursing and Management College, Yilan County 266, Taiwan; ^2^Department of Emergency and Critical Care, Cheng-Hsin General Hospital, Taipei 112, Taiwan; ^3^Department of Medicine, College of Medicine, Fu Jen Catholic University, New Taipei City 24205, Taiwan; ^4^Division of Chest Medicine, Department of Internal Medicine, Shin Kong Wu-Ho-Su Memorial Hospital, Taipei 11101, Taiwan; ^5^Division of Thoracic Surgery, Department of Surgery, Chi-Mei Foundation Medical Center, Tainan 71004, Taiwan; ^6^Department of Pathology, Cardinal Tien Hospital, New Taipei City 23148, Taiwan

## Abstract

Nanotitanium dioxide particle (nTiO_2_) inhalation has been reported to induce lung parenchymal injury. After inhalation of nTiO_2_, we monitored changes in 5-lipoxygenase, endothelial nitric oxide synthase (eNOS), and inducible nitric oxide synthase (iNOS) mRNA in rat lung tissue. Lung function parameters include specific airway resistance (SRaw), peak expiratory flow rate (PEF), functional residual capacity (FRC), and lung compliance (Cchord); blood white blood cell count (WBC), nitric oxide (NO), hydrogen peroxide, and lactic dehydrogenase (LDH); and lung lavage leukotriene C4, interleukin 6 (IL6), tumor necrotic factor **α** (TNF**α**), hydroxyl radicals, and NO. Leukotriene receptor antagonist MK571 and 5-lipoxygenase inhibitor MK886 were used for pharmacologic intervention. Compared to control, nTiO_2_ exposure induced near 5-fold increase in 5-lipoxygenase mRNA expression in lung tissue. iNOS mRNA increased while eNOS mRNA decreased. Lavage leukotriene C4; IL6; TNF**α**; NO; hydroxyl radicals; and blood WBC, NO, hydrogen peroxide, and LDH levels rose. Obstructive ventilatory insufficiency was observed. MK571 and MK886 both attenuated the systemic inflammation and lung function changes. We conclude that inhaled nTiO_2_ induces systemic inflammation, cytokine release, and oxidative and nitrosative stress in the lung. The lipoxygenase pathway products, mediated by oxygen radicals and WBC, play a critical role in the obstructive ventilatory insufficiency induced by nTiO_2_.

## 1. Introduction

Nanotechnology has assumed an important role in our lives as its application becomes increasingly prevalent across all segments of industry. By August, 2009, statistics revealed more than 1,000 consumer products available utilizing nanotechnology. It can be found in food and beverage preparations, gardening, health, clothing, cosmetics, automobile, electronics, toys, appliances, and many other products. However, as nanotechnology intertwines itself with our lives, its impact on human health has come into question. How to minimize harmful exposure to nanoparticles both in the workplace and the home environment is becoming the focus of study in many research institutions.

Increased nanoparticle concentrations have been found in the air of storage and packaging facilities of nanoproducts, raising question of occupational health hazard [1, 2]. High heat environment such as welding is particularly likely to cause the release of nanoparticle smoke. Nanoparticles released from exhaust pipes of motor vehicles can cause lung inflammation and fibrosis [3]. It also induces airway hypersensitivity [4]. Therefore, nanoparticles are potentially harmful. They can be released accidentally into the environment as part of the production process or during waste disposal. Routes of exposure include inhalation, skin contact, or ingestion. Occupational risks aside, exposure to nanoparticles in the home environment is also an increasing concern.

Research regarding the toxicity of nanocomposites has been done on only the more common nanomaterials, including carbon black, carbon nanotubes, nanozinc oxide, nanocalcium carbonate, polystyrene, nanosilica, and nanotitanium dioxide (nTiO_2_). Nanotitanium dioxide is commonly used in paint pigment, glass, wall paper, and ceramic tiles. It has often been added to paint as a photocatalyst for disinfection, detoxification, or self-cleaning purposes. Its application is widespread. There are numerous publications in recent years on the harmful effects of nanopowders on both cellular and organismal levels [5]. Animal studies have shown that inhalational exposure to nanoparticles can lead to emphysema, alveolar macrophage aggregation, diffuse alveolar damage, proliferation of type II alveolar epithelial cells, and epithelial apoptosis in rats [6]. Other studies reported exposure to nTiO_2_ modulates asthma-like hypersensitivity and inflammatory response in lungs [7]. Dispersion of nanoparticles is known to cause inflammation and injury to multiple organs, including lung, kidney, spleen, and liver [8]. However, up to now there have not been reports of its impact on formal pulmonary function testing.

In our study a dry powder insufflator was used to instill different concentrations of nTiO_2_ into rat lungs. We recorded the changes in pulmonary function, the level of nitrosative and oxidative stress, cytokines in both blood and bronchoalveolar lavage fluid (BALF), and the serum lactate dehydrogenase (LDH) and white blood cell (WBC) concentrations after insufflation to further our understanding of the effect of nTiO_2_ exposure on pulmonary function and systemic inflammation. Our study also provided dosing information for future animal experiments.

## 2. Materials and Methods

### 2.1. Preparation of Animals and Experimental Design

Thirty-one male Sprague-Dawley rats (300 to 350 g, pathogen-free) acquired from the National Animal Center, Taiwan, were housed in a controlled environment at 22 ± 1°C under a 12 h/12 h light/dark cycle. Food and water were available *ad libitum*. Care and use of the animals were in accordance with the National Animal Center guidelines. Rats fasted overnight with free access to water for 12 hr before the experiment.

Animals were randomly divided into four groups. In the MK571 leukotriene receptor antagonist group (*n* = 8), rats received MK571 (Sigma 7571) (0.1 mg/kg) by intraperitoneal administration 12 hr before nTiO_2_ exposure. In the MK886 leukotriene synthase inhibitor group (Sigma M2692) (*n* = 8), rats received MK886 (0.1 mg/kg) by intraperitoneal administration 12 hr before nTiO_2_ exposure. In the control group (*n* = 8), rats were given no pharmacological treatment before nTiO_2_. Rats in the sham group (*n* = 7) were prepared in the same manner as the control group, except they were not subjected to nTiO_2_ exposure. We analyzed the inflammatory responses of the animals by measuring the changes in blood and lavage WBC, oxygen radicals, NO, and LDH. Lavage leukotriene C4, IL6, and TNF*α* concentrations were also measured. Lung function was evaluated by measuring changes in functional residual capacity (FRC), airway resistance (SRaw), peak expiratory flow rate (PEF), and lung compliance (Cchord).

### 2.2. nTiO_2_ Exposure Model

The experimental animal was first placed in a chamber for induction of anesthesia with isoflurane. After areflexia was confirmed, it was secured on a working platform. A mask was placed over its nose and isoflurane 2% was given through the mask at 2 mL/min continuously. A small animal laryngoscope was then inserted into the trachea. Oral secretion was suctioned. A dry powder insufflator (Model DP-4, Penn Century, Wyndmoor, USA) was used to administer a body weight-adjusted dose (4 mg/kg, 10 mg/kg, and 20 mg/kg) of nTiO_2_ (titanium dioxide P25 21 nm nanograde, Anatase-80%, and Rutile-20% from SIGMA) directly into the trachea in one push.

### 2.3. Functional Residual Capacity and Lung Compliance Measurements

Lung capacity was measured by total body plethysmography equipped with software-operated solenoid valves connecting the tracheal catheter to outside air or positive and negative pressure reservoirs (Buxco Electronics, Inc., Wilmington, USA). Rats were anesthetized with pentobarbital (50 mg/kg I.P.). The right femoral artery and vein were cannulated for blood pressure monitoring and saline administration. Intubated rats were placed in the right recumbent position for plethysmography and three lung inflations to 30 cmH_2_O were delivered before each set of measurements. Following three lung inflations and return to normal tidal breathing, the airway opening was occluded at end-expiratory lung volume for 8 s, while the changes in airway pressure and thoracic volumes were recorded during inspiratory efforts. FRC and Cchord were computed from these data and the ambient barometric pressure by Boyle's Law method (Pulmonary Maneuvers Software, Buxco Electronics).

### 2.4. Lung Function of Airway Resistance and Peak Airflow Rate Measurements

Respiratory function in the conscious rat was assessed by two-chamber whole body plethysmography (Buxco Electronics, Inc., Wilmington, USA.). SRaw and PEF before and after titanium dioxide exposure treatment were determined. Each chamber of the plethysmograph was connected to a differential pressure transducer linked to an amplifier system (Buxco Electronics model number PLY-3023); and respiratory parameters were captured and analyzed by the Biosystem XA data acquisition system. Before and after sham or titanium dioxide exposure treatment, the animal was placed in the plethysmography chamber; SRaw and PEF were recorded [9].

### 2.5. Bronchoalveolar Lavage

Bronchoalveolar lavage was performed 5 min after the conclusion of the experiment. A 2.5 mL aliquot of warm saline (37°C) was introduced into the trachea and then gently suctioned out with a 5 mL syringe. Average return of fluid was 1.5 ± 0.3 mL per 2.5 mL aliquot. Lavage samples were cooled to 4°C until the conclusion of the experiment and then centrifuged (1.300 rpm) at that temperature for 10 min. The supernatants were saved for mediator assays.

### 2.6. Methyl Guanidine Measurement

As the formation of methyl guanidine (MG) is an index of hydroxyl radical production [10], MG in BALF was measured to reflect titanium dioxide-induced hydroxyl radical production. A spectrofluorometer (Jusco 821-FP, Hachioji, Japan) measured the fluorescence spectra for emission maximum at 500 nm and excitation maximum at 395 nm. Lavage samples were diluted with water (1 : 100) and measured. The assay was calibrated with authentic MG (Sigma M0377, St. Louis, USA). Standard methyl guanidine concentrations (0, 50, 100, 150, and 200 mg/mL) were used to setup a standard curve (MG concentration = 369.0 × absorption value + 12.3; *R* ^2^ = 0.95).

### 2.7. Hydrogen Peroxide Measurements

Blood concentrations of hydrogen peroxide were measured after titanium dioxide exposure using a free oxygen radicals test (FORT, Callegari, FORM OX, Italy) according to the manufacturer's manual. The radical species produced were measured by 20 *μ*L blood samples. Samples interact with a reagent that forms a radical molecule which is evaluable by spectrophotometer at 505 nm.

### 2.8. Measurement of Nitrite/Nitrate by High-Performance Liquid Chromatography

Levels of nitrite/nitrate (the metabolites of nitrogen oxide) in the blood and lung lavage fluid were determined by high-performance liquid chromatography. This method has a sensitivity of 1 pmol for each anion. As little as 0.05–0.1 mL of sample volume is required and linearity is observed up to 60 nmol for each anion. Before injection into the chromatographic system (ENO-20, EicomNox Analyzer, Kyoto, Japan), the samples were diluted and subjected to suitable cleanup procedures, and serum samples were deproteinized by ultrafiltration through membranes with a molecular mass cutoff of 3.000. The samples were separated on a strong anion-exchange column (Spherisorb SAX, 250 × 4.6 mm I.D., 5 *μ*m) followed by two on-line postcolumn reactions. The first involved nitrate reduction to nitrite on a copper-plated cadmium-filled column; the second reaction involved a diazotization-coupling reaction between nitrite and the Griess reagent (0.05% naphthylenediamine dihydrochloride plus 0.5% sulphanilamide in 5% phosphoric acid). The absorbance of the chromophore was read at 540 nm.

### 2.9. Leukotriene C4 Measurement

Leukotriene C4 concentration in lung lavage fluid was measured using a leukotriene C4 EIA kit. Supernatants separated from the centrifuged lavage fluid were measured by enzyme immunoassay according the manufacturer's instruction using reagent purchased from Cayman Chemical Company (Catalog number 52021strip plate, Ann Arbor, USA).

### 2.10. IL6 Measurement by ELISA

IL6 concentration in lung lavage samples was measured separately with an enzyme-linked immunosorbent assay kit (Invitrogen, Woburn, USA). All samples were stored at −70°C before testing. All reagents, samples, and working standards were brought to room temperature and prepared according to the manufacturer's directions. The ELISA was likewise performed following the manufacturer's instructions. Each sample was performed in duplicates and read by an automated ELISA reader at a wavelength of 450 nm.

### 2.11. TNF*α* Measurement by ELISA

TNF*α* concentration in lung lavage samples was measured separately by an enzyme-linked immunosorbent assay kit (Endogen, Woburn, USA). All samples were stored at −70°C before testing. All reagents, samples, and working standards were brought to room temperature and prepared according to the manufacturer's directions. The ELISA was likewise performed following the manufacturer's instructions. Each sample was performed in duplicates and read by an automated ELISA reader at wavelengths of 450/540 nm.

### 2.12. WBC Count by Hemocytometry

WBC counts were performed by hemocytometry at the beginning and the end of the experiment.

### 2.13. LDH Determination

Blood samples were kept at 4°C and centrifuged (3000 ×g, 5 min), and 50 *μ*L of the supernatant was withdrawn and analyzed for LDH in a dry chemistry analyzer (Fuji Dri-Chem 3000, Japan).

### 2.14. mRNA Expressions of 5-Lipoxygenase, eNOS, and iNOS

Isolation of mRNA from lung tissue was performed with an mRNA Isolation Kit (QIAGEN RNeasy kits, QIAGEN Inc., Valencia, USA.). The mRNA isolated from each lung tissue sample was reverse transcribed to cDNA following the manufacturer's recommended procedures.

### 2.15. Real-Time PCR

PCR primers and TaqMan-MGB probes (Table 1) were designed using Primer Express V.2.0 software (Applied Biosystems Inc., Foster, USA) based on the sequences from GenBank. TaqMan-MGB probes were labeled with 6-carboxy-fluorescein as the reporter dye. Real-time PCR was performed in a two-step process.

In the first step, sample RNA (100 ng) was reverse transcribed with 50 ng random hexamers in a volume of 20 *μ*L using 200 U of Superscript III reverse transcriptase and 40 U of RNaseOUT recombinant RNase inhibitor (both from Invitrogen, Carlsbad, USA). In the second step, real-time PCR was carried out in a MicroAmp Optical 96-well plate using TaqMan Master Mix (Applied Biosystems Inc., Foster, USA), with 5 *μ*L l cDNA in each well. PCR reactions were monitored in real time using the ABI PRISM 7000 Sequence Detector (Applied Biosystems Inc.). The thermal cycling conditions for real-time PCR were (a) 50°C for 2 min, (b) 95°C for 10 min, and (c) 40 cycles of melting (95°C, 15 sec) and annealing/extension (60°C, 60 sec). The relationship between the initial amount *A* of target present and the amount *Xn* of DNA produced after *n* PCR cycle can be expressed as *Xn* = *A* × (1+*E*)^*n*^, where *E* is the amplification efficiency of one PCR step. Threshold cycle (Ct) indicates the fractional cycle number at which the amount of amplified target reaches a fixed threshold. The variation in gene expression of candidate genes A and B is shown by ΔCt. The relative gene expression of target, normalized to an endogenous reference (18 s rRNA; supplied by Applied Biosystems Inc.) and relative to a calibrator, was determined by 2-ΔΔCt in various tissues. Less ΔCt means higher target mRNA expression before amplified.

### 2.16. Preparation of Pathologic Specimens and Stains

The lungs were removed immediately from rats in the nTiO_2_ group following the experiment for histological studies. The lungs were fixed in 10% neutral formalin for 24 hr at room temperature. Tissue samples were taken from the central and lower portions of the right lower lobe. After dehydration and cleaning, specimens were embedded in paraffin and cut into 4 *μ*m thick slices using a microtome. Staining was performed using hematoxylin and eosin (H&E) and Masson trichrome stain, which reacts strongly with connective tissues.

### 2.17. Data Analysis

Data were expressed as means ± SEM. Comparisons among 4 groups were made by one-way ANOVA and Scheffe's comparison. Comparisons within each group for a given parameter were made using paired Student's *t* tests. *P* values < 0.05 were considered statistically significant.

## 3. Results and Discussion

**Figure 1:**
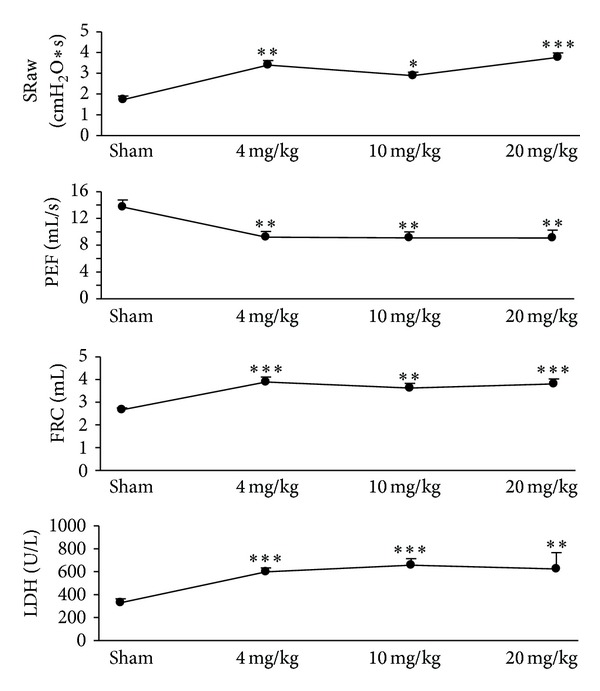
Changes in lung mechanics in rats induced by different doses of nanotitanium dioxide inhalation. After-inhalation significant increases in specific airway resistance (SRaw) and functional residual capacity (FRC) were observed; and significant decreases in peak expiratory flow (PEF) were noted. Lung parenchymal damage as represented by lactate dehydrogenase (LDH) level rose after-exposure (*P < 0.05; **P < 0.01; ***P < 0.001).

Pilot tests were run to determine the optimal dosing of nTiO_2_. After nTiO_2_ exposure, significant increases in specific airway resistance (SRaw) and functional residual capacity (FRC) were observed and significant decreases in peak expiratory flow (PEF) were noted. Lung parenchymal damage as represented by lactate dehydrogenase (LDH) level rose after-exposure as well. However, there was no difference in response in the three dosing groups (4 mg/kg, 10 mg/kg, and 20 mg/kg) (Figure 1). As a result, only the lowest concentration (4 mg/kg) of nTiO_2_ was used in all subsequent experiments.

**Figure 2:**
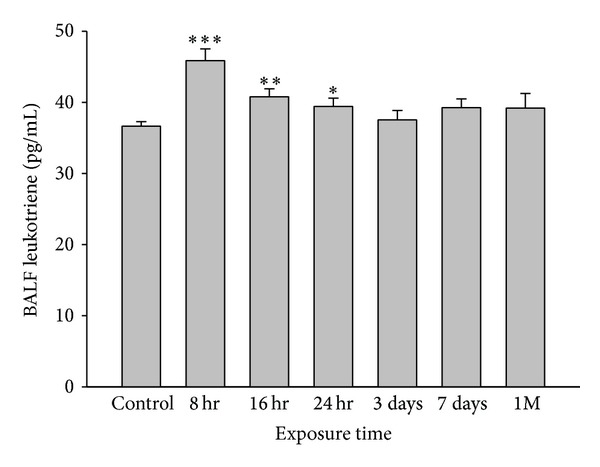
Kinetic changes in leukotriene C4 concentration in bronchoalveolar lavage fluid (BALF) after nanotitanium dioxide inhalation. The peak leukotriene C4 level was observed 8 hr after-exposure. Therefore, in all subsequent experiments, measurements were taken at 8 hr after-exposure (*P < 0.05; **P < 0.01; ***P < 0.001).

After choosing the optimal dose, tests were run to determine the ideal duration of the experimental protocol. Leukotriene C4 level peaked at 8 hr after-nTiO_2_ exposure. Therefore, in all subsequent experiments, measurements were taken at 8 hr after-exposure (Figure 2).

**Figure 3:**
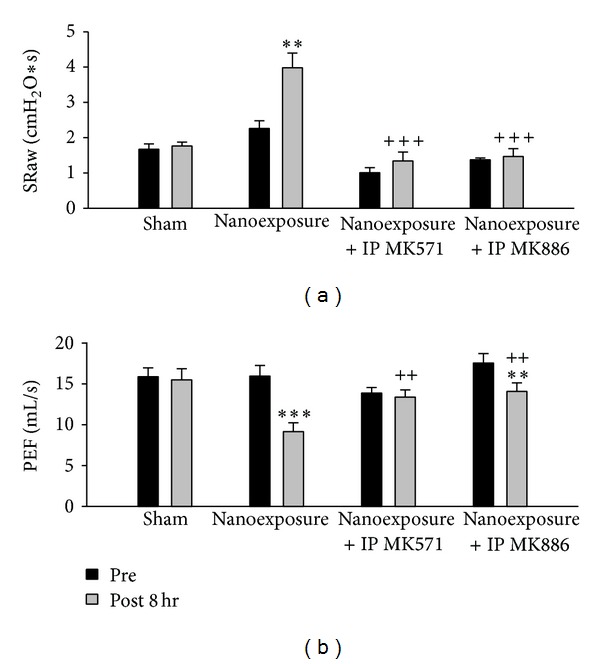
Aerosolized nanoparticles of titanium dioxide (4 mg/kg) induced significant increase of specific airway resistance (SRaw), significant decrease of peak expiratory flow rate (PEF), and the effects of leukotriene receptor antagonist (MK571) and leukotriene synthase inhibitor (MK886) before-treatment. (**P < 0.01; ***P < 0.001; ^++^ *P* < 0.01; ^+++^ *P* < 0.001. ∗ denotes comparison before- and after-exposure; + denotes comparison with nanoexposure control.)

SRaw was significantly increased after inhalation of nTiO_2_ compared to the sham group. Pretreatment with either leukotriene receptor antagonist MK571 or leukotriene synthase inhibitor MK886 attenuated the increase in SRaw. PEF was significantly decreased after inhalation of nTiO_2_. Pretreatment with either MK571 or MK886 attenuated the drop in PF induced by nTiO_2_ inhalation (Figure 3).

**Figure 4:**
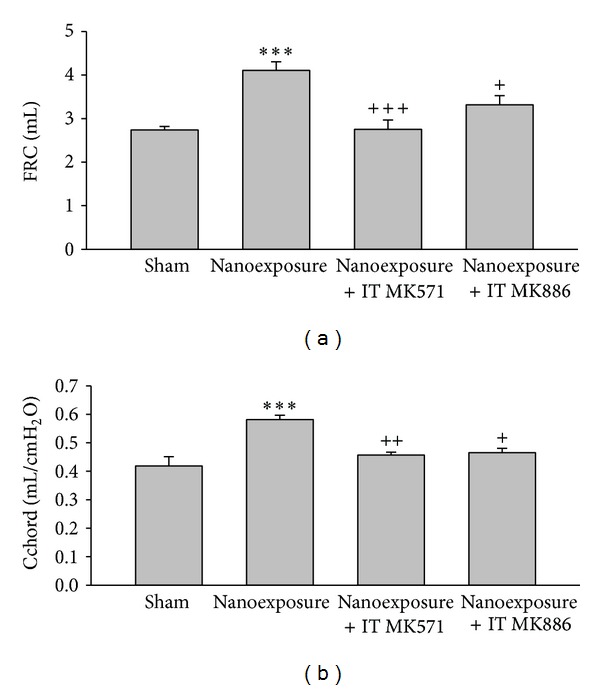
Aerosolized nanoparticles of titanium dioxide (4 mg/kg) induced significant increases of functional residual capacity (FRC) and lung compliance (Cchord) and the effects of leukotriene receptor antagonist (MK571) and leukotriene synthase inhibitor (MK886) before-treatment. (***P < 0.001; ^+^ *P* < 0.05; ^++^ *P* < 0.01; ^+++^ *P* < 0.001. ∗ denotes comparison before- and after-exposure; + denotes comparison with nanoexposure control.)

Inhalation of nTiO_2_ significantly increased the Cchord. Pretreatment with MK571 or MK886 abrogated the rise in Cchord. Likewise, nTiO_2_ significantly increased the FRC. Pretreatment with MK571 or MK886 abrogated the increase in FRC (Figure 4).

**Figure 5:**
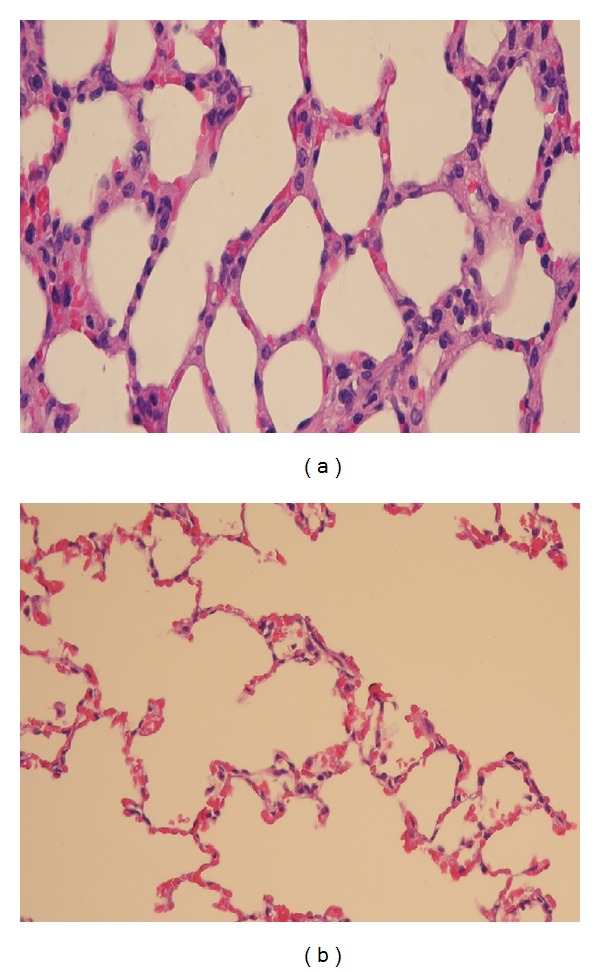
Lung tissue samples from the central and lower portions of the right lower lobe stained with hematoxylin and eosin (H&E) and Masson trichrome stain: (a) rat lung taken from the sham group and (b) rat lung from the nTiO_2_ exposure control group.

**Figure 6:**
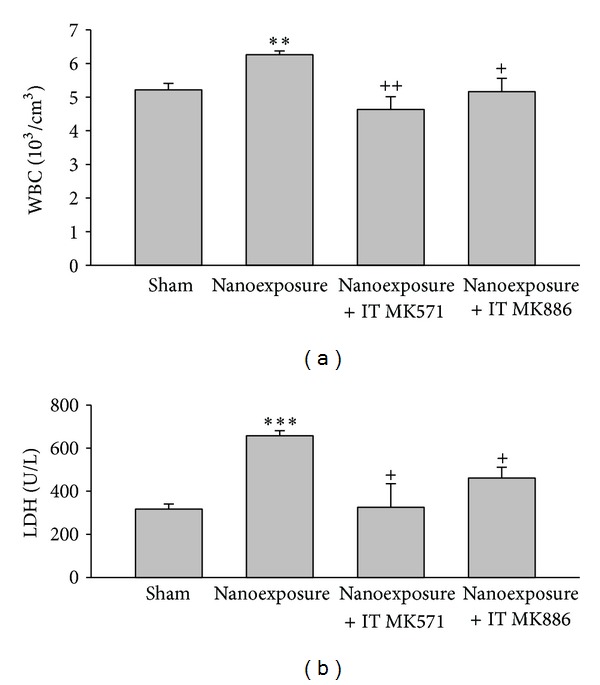
Aerosolized nanoparticles of titanium dioxide (4 mg/kg) induced significant increases in white blood cell count (WBC) and lactic dehydrogenase (LDH) and the effects of leukotriene receptor antagonist (MK571) and leukotriene synthase inhibitor (MK886) before-treatment. (**P < 0.01; ***P < 0.001; ^+^ *P* < 0.05; ^++^ *P* < 0.01. ∗ denotes comparison before- and after-exposure; + denotes comparison with nanoexposure control.)

**Figure 7:**
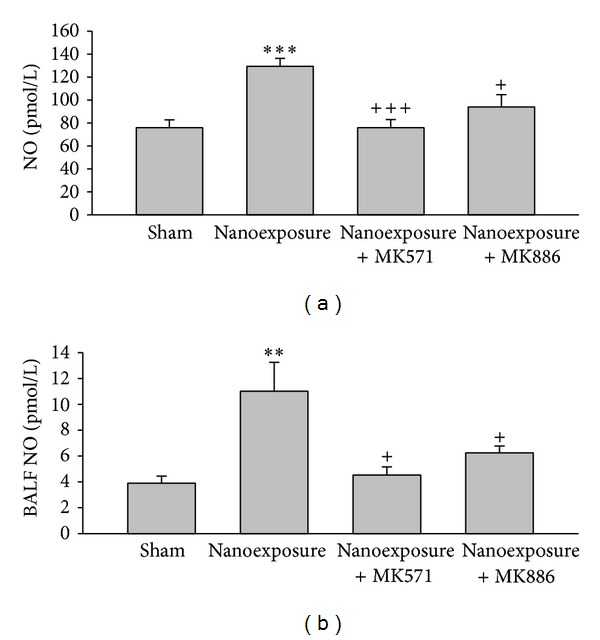
Aerosolized nanoparticles of titanium dioxide (4 mg/kg) induced significant rise of nitric oxide (NO) in blood (a) and bronchoalveolar lavage fluid (BALF) (b) and the effects of leukotriene receptor antagonist (MK571) and leukotriene synthase inhibitor (MK886) before-treatment. (**P < 0.01; ***P < 0.001; ^+^ *P* < 0.05; ^+++^ *P* < 0.001. ∗ denotes comparison before- and after-exposure; + denotes comparison with nanoexposure control.)

**Figure 8:**
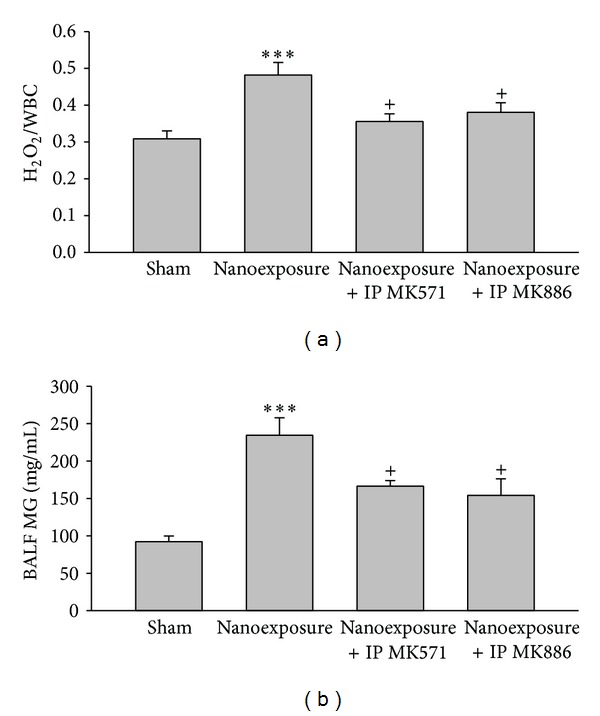
Aerosolized nanoparticles of titanium dioxide (4 mg/kg) induced significant oxygen radical release by WBC in blood (H_2_O_2_/WBC) (a) and bronchoalveolar lavage fluid (BALF) represented by methylguanidine (MG) (b) and the effects of leukotriene receptor antagonist (MK571) and leukotriene synthase inhibitor (MK886) before-treatment. (***P < 0.001; ^+^ *P* < 0.05. ∗ denotes comparison before- and after-exposure; + denotes comparison with nanoexposure control.)

**Figure 9:**
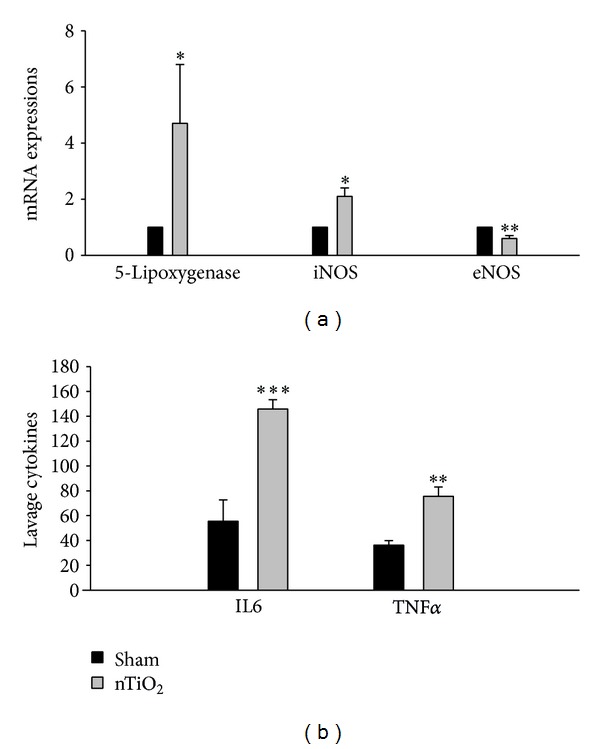
mRNA expressions of 5-lipoxygenase, iNOS, and eNOS after TiO_2_ exposure. The results showed upregulation of 5-lipoxygenase and iNOS expressions and downregulation of eNOS expression in rat lung after TiO_2_ exposure. nTiO_2_ exposure also induced significant increases in IL6 and TNF*α* concentrations in BALF. (*P < 0.05, **P < 0.01, and ***P < 0.001).

Pathologic examination of lung tissue showed that nTiO_2_ induced emphysematous changes with increased size of alveoli and alveolar wall destruction (Figure 5(b)), when compared with the sham rat lung (Figure 5(a)). Nano-TiO_2_ exposure induced significant increases in serum LDH and WBC. Pretreatment with MK571 or MK886 abolished the effect (Figure 6). Nano-TiO_2_ inhalation caused an increase in nitrite/nitrate concentration in both BALF and blood, which was prevented by pretreatment with either MK571 or MK886 (Figure 7). Similar to its effect on nitrosative stress, nTiO_2_ inhalation resulted in a rise in hydrogen peroxide and the byproducts of hydroxyl radical in blood and BALF. Pretreatment with either MK571 or MK886 attenuated the rise (Figure 8). Increased respiratory burst and nitric oxide burst are both associated with nTiO_2_-induced lung inflammation in our model. Real-time PCR data showed that nTiO_2_ resulted in an increase in iNOS expression (1.00 ± 0.00 versus 2.13 ± 0.30, *P* < 0.05) but not eNOS expression (1.00 ± 0.00 versus 0.61 ± 0.07, *P* < 0.01) (Figure 9). Nano-TiO_2_ exposure also induced an almost 5-fold increase in 5-lipoxygenase mRNA expression in lung tissue compared to control (4.7 ± 2.1 versus 1.0 ± 0.0, *P* < 0.05). The interaction among white blood cells, oxygen radicals, arachidonic acid, and cytokines is known to be complex. In this study we showed that nTiO_2_ inhalation induced an increase in IL6 and TNF*α* concentrations in BALF, both well-known cytokines in the inflammatory response.

As nanoparticle products become more and more prevalent in the market place, their potential harm to humans has become the focus of intense research. Previous studies on nanoparticles and their effects focus mostly on their ability to trigger inflammatory responses [11, 12]. Even though there is more than one route of exposure, the respiratory tract is thought to be the most important, especially in the work place through handling and manufacturing of dispersible nanoparticles.

We see ample evidence of inflammation associated with nanoparticle exposure through the respiratory tract, in terms of elevated WBC, aggregation of alveolar macrophages and neutrophils, and increased cytokines and LDH in the BALF [11, 13, 14]. The degree of inflammation can be affected by the size, concentration, and shape of the nanoparticle, which would alter its surface area, which has emerged to be an important predictor of toxicity [15–18]. As part of the possible mechanisms within the systemic inflammatory response, nanoparticles are known to cause an increase in lipid peroxidation, antioxidant activity, and cellular oxidative stress in general [11, 19]. It is thought that it in fact modulates intracellular calcium concentrations, activation of transcriptional factors associated with inflammation, and cytokine production through the generation of reactive oxygen species [20]. It has also been known to cause structural damage to the mitochondria and the redox cycle; thus, further increases oxidative stress [21].

In addition to proinflammatory actions systemically and locally in the lung, nanoparticle inhalation also causes histological changes in the lung. Similar to the spectrum of histological changes seen in other inflammatory conditions in the lung such as chronic obstructive pulmonary disease, nanoparticle exposure has been known to cause alveolar wall destruction, emphysematous changes, and fibrosis in the lung [6]. There are reported cell proliferations in the bronchi and bronchioles following nanoparticle inhalation [22, 23] and an accumulation of alveolar macrophages is also observed [4, 24].

Alveolar macrophages play an important role in nanoparticle-induced pulmonary toxicities. The phagocytotic ability of macrophages is impaired following high-dose nTiO_2_ inhalation [25], and its sensitivity to the chemotactic agent complement 5a is enhanced [14], facilitating further accumulation of inflammatory cells. nTiO_2_ are known to generate lipid mediators in alveolar macrophages [26]. The oxidation of lipids can be channeled down either the cyclooxygenase or the lipoxygenase pathway. The cyclooxygenase pathway produces the prostaglandins and the lipoxygenase pathway the leukotrienes. The production of these lipid mediators can be affected by inhaled materials; for instance, tobacco smoke has been shown to inhibit the production of leukotriene B4 by human alveolar macrophages [27]. The leukotrienes are produced by various cells and tissues. Their known activities include contraction of bronchial smooth muscles, stimulation of vascular permeability, and attraction and activation of leukocytes [28]. Leukotrienes are known to be involved in obstructive pulmonary insufficiency [29, 30]. In fact, leukotriene antagonists are established therapeutic agents for asthma.

Our study demonstrated that nTiO_2_ inhalation triggered systemic inflammation, resulting in elevated serum WBC, LDH, IL6, and TNF*α*. It also caused an increase in both nitrosative and oxidative stress both systemically and locally in the lung. Our findings thus far validated previous reports. In addition, few studies reported on the functional effect on the lung resulting from nanoparticle exposure. Some studies reported an increase in airway resistance and a decrease in the peak expiratory flow rate as well as variable airway reactivity with different coatings of nTiO_2_ [7, 31]. In our study, we showed clear evidence of an increase in airway resistance, functional residual capacity, and lung compliance, as well as a decrease in peak expiratory flow rate following exposure. By pretreating the animals with either a leukotriene receptor antagonist (against LTD4) or a 5-lipoxygenase inhibitor (against LTB4 and LTC4), all of the inflammatory and pulmonary function parameter changes can be abrogated. This suggests that the lipoxygenase pathway is involved in nTiO_2_ related pulmonary inflammation and resultant obstructive pulmonary function changes.

## 4. Conclusions

We conclude from these experiments that nTiO_2_ exposure induces systemic inflammation and increases oxidative and nitrosative stress in the lung. The lipoxygenase pathway products might play critical roles in the obstructive ventilatory insufficiency induced by nTiO_2_ exposure.

## Figures and Tables

**Table 1:** Sequences of PCR primers for putative 5-lipoxygenase, eNOS, and iNOS target genes.

	Forward primer	Reverse primer
5-Lipoxygenase	TCTGGGTGCGTTCAAGTGACT	CCAGATGTGTGCGGAGAAGA
eNOS	CCGGGACTTCATCAATCAGTACTAT	CCTGAAGCCGCTGCTCAT
iNOS	TGCTTACAGGTCTACGTTCAAGACAT	CGGCCACCAGCTTCTTCA
